# SCA14-Associated PKCγ-G118D Mutant Exhibits a Detrimental Effect on Cerebellar Purkinje Cell Dendritic Growth

**DOI:** 10.3390/ijms26083688

**Published:** 2025-04-14

**Authors:** Qin-Wei Wu, Kejian Wang, Josef P. Kapfhammer

**Affiliations:** 1 School of Life Sciences, Anhui University, Hefei 230601, China; 2 Key Laboratory of Human Microenvironment and Precision Medicine of Anhui Higher Education Institutes, Anhui University, Hefei 230601, China; 3 Institute of Anatomy, Department of Biomedicine, University of Basel, 4056 Basel, Switzerland

**Keywords:** spinocerebellar ataxia, PKCγ, cerebellar Purkinje cells, protein kinase C gamma, Purkinje cell dendritic development, Purkinje neurons

## Abstract

Spinocerebellar ataxia (SCA), an autosomal dominant neurodegenerative condition, is marked by a gradual deterioration of cerebellar function. To date, more than 40 distinct SCA subtypes have been identified, with some attributed to CAG repeat expansions and others to point mutations or deletions. Among these, spinocerebellar ataxia type 14 (SCA14) stems from missense mutations or deletions within the *PRKCG* gene, encoding protein kinase C gamma (PKCγ), a pivotal signaling molecule abundant in Purkinje cells. Despite its significance, the precise mechanisms underlying how genetic alterations trigger Purkinje cell malfunction and degeneration remain elusive. Given the prominent role and high expression of PKCγ in Purkinje cells, SCA14 presents a unique opportunity to unravel the underlying pathogenesis. A straightforward hypothesis posits that alterations in the biological activity of PKCγ underlie the disease phenotype, and there are hints that mutated PKCγ proteins exhibit altered enzymatic function. Our prior research focused on the PKCγ-G118D mutation, commonly found in SCA14 patients, located in the regulatory domain of the protein. While cellular assays demonstrated enhanced enzymatic activity for PKCγ-G118D, transgenic mice carrying this mutation failed to exhibit suppressed dendritic development in cerebellar cultures, raising questions about its impact within living Purkinje cells. One hypothesis is that endogenous PKCγ might interfere with the expression or effect of PKCγ-G118D. To further investigate, we leveraged CRISPR-Cas9 technology to generate a PKCγ knockout mouse model and integrated it with an L7-based, Purkinje cell-specific transfection system to analyze the effects of G118D protein expression on the dendritic morphology of developing Purkinje cells. Our findings reveal that, utilizing this approach, PKCγ-G118D exerts a detrimental effect on Purkinje cell growth, confirming its negative influence, indicating that the potential of the G118D mutation to contribute to SCA14 pathogenesis.

## 1. Introduction

The diverse spectrum of Spinocerebellar ataxias (SCAs) comprises a set of inherited disorders arising from genetic mutations across various genes. Notably, a prevalent subset of these conditions is attributed to poly-glutamine repetitions within the corresponding genetic sequences [1,2,3,4,5,6,7,8,9,10,11,12]. Additionally, certain SCA forms, such as SCA14, stem from point mutations within specific genes, notably the *PRKCG* gene, which encodes protein kinase C gamma (PKCγ). SCA14 exhibits an autosomal dominant inheritance pattern and is clinically characterized by a progressive decline in cerebellar function, along with the degeneration of Purkinje cells. PKC, a superfamily of serine/threonine kinases, plays a pivotal role in cellular signaling across numerous cell types [13,14,15,16]. Within this family, PKCγ belongs to the classical subclass and possesses a unique structural architecture, comprising four conserved domains and five variable regions. The activation of PKCγ is intricately regulated by two regulatory domains: the C1 domain, which binds to diacylglycerol (DAG) and phorbol esters, and the C2 domain, which interacts with Ca^2+^. The catalytic core of PKCγ encompasses the C3 and C4 regions, where the C3 harbors an ATP-binding site, while the C4 constitutes the primary catalytic site, displaying a high degree of conservation both within PKC isoforms and across species [17,18]. In the context of SCA14, a preponderance of mutations has been identified within the regulatory C1 domain of PKCγ, with a minority also occurring in the C2 domain and the catalytic C3 and C4 domains [13,17,18]. However, the precise mechanisms underlying how these diverse mutations give rise to the SCA14 phenotype remain elusive. The pivotal role of PKCγ activity extends to shaping the dendritic architecture of Purkinje cells, a crucial aspect of their development. Studies utilizing cerebellar Purkinje cell cultures have revealed a dual effect: PKC signaling activation significantly impedes Purkinje cell dendritic growth and maturation, whereas its inhibition fosters dendritic elaboration. Consequently, the morphological profile of developing Purkinje cells serves as a proxy for endogenous PKCγ activation levels, as robust or sustained activation leads to Purkinje cells with markedly attenuated dendrites [18]. The dendritic perturbations observed post-PKC activation align with the hypothesis that SCA14 pathogenesis may stem, at least partially, from a gain-of-function scenario involving overly potent or constitutively active PKCγ. This notion is reinforced by experimental evidence demonstrating that numerous PKCγ mutations enhance its catalytic activity in vitro and cellular assays [18,19]. Conversely, there are reports suggesting that C1B domain mutations in PKCγ may impair function by diminishing DAG binding, while other studies propose that SCA14 mutations disrupt structural integrity of PKCγ, promoting instability or aggregation, ultimately leading to loss of function [18,19]. Intriguingly, despite these contrasting mechanisms, Purkinje cells transfected with mutant PKCγ consistently exhibit abnormal dendritic development, regardless of aggregate formation [17,18]. Collectively, these findings hint at the possibility that the heightened kinase activity observed in cellular assays may contribute to the underlying pathology of SCA14, though the precise mechanisms remain multifaceted and require further elucidation.

In our previous investigations, we observed no discernible dendritic alterations in Purkinje cells derived from transgenic mice harboring the C1 domain G118D mutation in cerebellar Purkinje cell cultures [18]. A plausible explanation for this observation is that the G118D-mutated protein may be expressed at low levels in the presence of endogenous PKCγ in these transgenic mice, insufficient to elicit a phenotypic response. Prompted by this observation, we delved deeper into the question of whether the G118D mutation, known to affect Purkinje cell dendritic development, could potentially underlie SCA14 pathogenesis. To disentangle the effects of endogenous PKCγ, we utilized CRISPR/Cas9 technology to generate PKCγ knockout mice and examined the impact of both G118D mutations and wild-type PKCγ on Purkinje cell dendritic development in dissociated cerebellar cultures, following transfection with an L7 promoter-driven expression plasmid in the PKCγ knockout background. Our results reveal that the G118D mutation, when expressed in the absence of endogenous PKCγ, is capable of inducing inhibition of dendritic growth in Purkinje cells. In contrast, the introduction of wild-type PKCγ into PKCγ-deficient Purkinje cells did not significantly alter their dendritic growth. These findings underscore the potential of the G118D mutation to contribute to SCA14 pathogenesis, independently of the presence of endogenous PKCγ.

## 2. Results

### 2.1. Purkinje Cell-Specific Human PKCγ Expression in Cerebellar Purkinje Neuron Cultures from PKCγ Knockout Mouse Model

**Figure 1:**
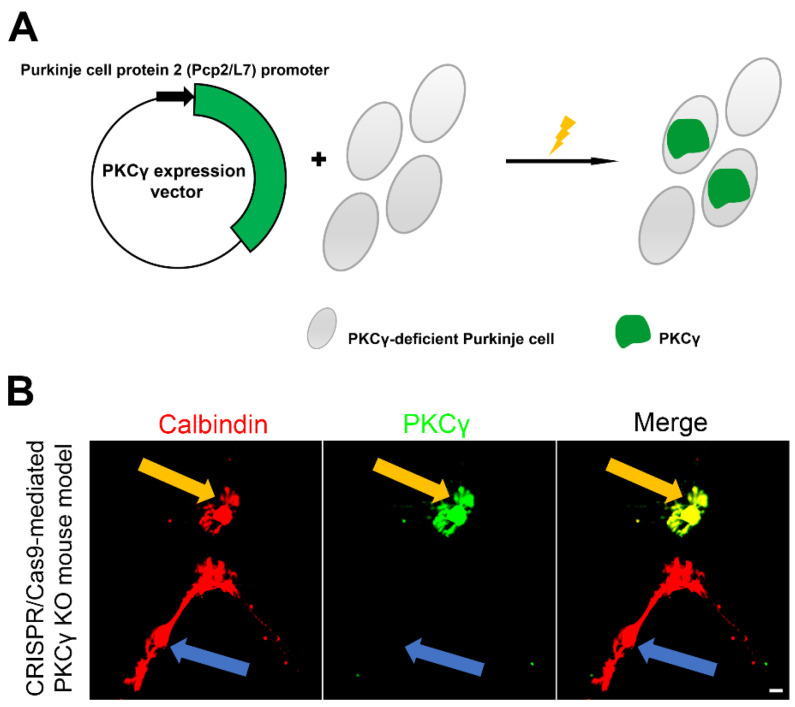
Purkinje-specific human PKCγ expression. (**A**) A schematic view of the human PKCγ expression construct specific to Purkinje cells, when introduced into PKCγ-deficient Purkinje cells, is shown, with the yellow symbol indicating the standard procedure for electroporating plasmids into cells; (**B**) Two Purkinje cells identified by the marker Calbindin (red) after two weeks in primary cerebellar cell culture of PKCγ knockout mice. L7- PKCγ transfected Purkinje cell (yellow arrow) and a non-transfected Purkinje cell (blue arrow) can be observed and distinguished by anti-PKCγ in the dissociated culture of PKCγ knockout Purkinje cells. The scale bar is 20 μm.

To establish a durable, Purkinje cell-specific expression system for human PKCγ in a PKCγ-null background, we first cloned the human *PRKCG* gene sequence and inserted it into an expression vector, harnessing the Pcp2/L7 promoter specifically active in Purkinje cells (Figure 1A). These custom-designed expression plasmids were promptly transfected into freshly dissociated Purkinje neurons on the day of culture initiation, ensuring efficient delivery. Utilizing the Pcp2/L7 promoter, we successfully induced sustained expression of human PKCγ in Purkinje cells, with the transgene remaining active for at least two weeks. Immunostaining with anti-Calbindin, a reliable marker for Purkinje cells, confirmed the specificity of our expression system, revealing robust human PKCγ expression exclusively in these neurons. In dissociated cultures derived from our PKCγ knockout mouse model, we could clearly distinguish Purkinje cells expressing the exogenous human PKCγ through staining with anti-PKCγ antibodies. Notably, non-transfected Purkinje cells remained devoid of PKCγ expression, as evidenced by their negative staining (Figure 1B), underscoring the specificity and efficacy of our approach.

### 2.2. SCA14-Assoicated PKCγ-G118D Impedes Development of Cultured PKCγ Knockout Purkinje Neurons

**Figure 2:**
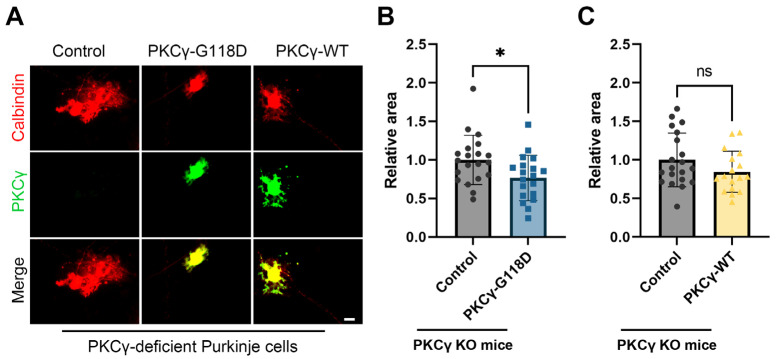
Purkinje-specific PKCγ-G118D expression inhibits Purkinje cell dendritic growth. (**A**) Representative images of PKCγ knockout Purkinje cells, non-transfected or transfected with the indicated plasmids; (**B**) The mean values of the Purkinje cell dendritic area were measured in three independent culture experiments of control and PKCγ-G118D groups. Control cells were non-transfected Purkinje cells from the same culture wells. Control: 1.00 ± 0.321, n = 20 cells; PKCγ-G118D: 0.77 ± 0.294, *n* = 19 cells; control vs. PKCγ-G118D; *p* = 0.0177 (*) in Mann–Whitney test; (**C**) The mean values of the Purkinje cell dendritic area were measured in three independent culture experiments of control and PKCγ-WT groups. Control: 1.00 ± 0.349, *n* = 20 cells; PKCγ-WT: 0.85 ± 0.268, *n* = 17 cells; Control vs. PKCγ-WT, not statistically significant (ns) in Mann–Whitney test; Data are expressed as mean ± SD. The scale bar is 20 μm.

To assess the differential impacts of the human disease-linked PKCγ-G118D mutant and its wild-type counterpart (designated as PKCγ-WT) on cerebellar Purkinje cells, we conducted an analysis focusing on the dendritic morphology of these cells after transfection with respective constructs. The human *PRKCG* gene, sourced from Origene in the pCMV6-XL4-PRKCG vector (Rockville, MD, USA), served as the starting point. Utilizing site-directed mutagenesis PCR, we generated a mutated *PRKCG* gene encoding the PKCγ-G118D protein and subsequently cloned this sequence into the pL7 vector, yielding the engineered pL7-PKCγ-G118D construct. This vector facilitated Purkinje cell-specific expression of the mutant protein. Given that previous studies have established that transfection with the pL7 vector alone does not alter Purkinje cell dendritic development compared to non-transfected controls [20], we compared the dendritic areas of Purkinje cells transfected with pL7-PKCγ-G118D and pL7-PKCγ-WT to those of non-transfected controls, all maintained under identical electroporation and culture conditions. The transfection with the PKCγ-G118D vector led to a significant decrease in the dendritic area, with the mean dendritic area of PKCγ-G118D-expressing Purkinje cells shrinking to 76.6% of controls. This finding underscores the significant inhibitory effect of the SCA14-associated PKCγ-G118D protein on dendritic growth (Figure 2A,B). Further, our analysis revealed that while the mean dendritic tree size of PKCγ-WT-expressing Purkinje cells was reduced to 84.5% of controls, this reduction did not attain statistical significance, indicating a relatively weak effect from PKCγ-WT (Figure 2A,C). Collectively, these results demonstrate that the PKCγ-G118D mutant exerts a detrimental influence on the development of cerebellar Purkinje cells in a PKCγ knockout context.

## 3. Discussion

Spinocerebellar ataxias (SCAs) represent a heterogeneous group of autosomal dominant, progressive disorders characterized by degeneration of the cerebellum. Coincidentally, numerous genes implicated as causative factors for SCAs, such as *SPTBN2*, *TRPC3*, *ITPR1*, *GRM1,* and *PRKCG*, have been found to play crucial roles in various stages of Purkinje cell development, including dendritic growth, differentiation, and maintenance. These genes encode proteins such as Beta-III spectrin, ITPR1, TRPC3, mGluR1, and PKCγ. For instance, Beta-III spectrin is critical for the organization of the dendritic tree and the development of dendritic spines in Purkinje cells. Studies on Beta-III spectrin knockout mice have revealed defects in ordered dendritic arborization, including a loss of monoplanar organization, decreased dendritic diameter, reduced density of dendritic spines, and a diminished number of synapses in Purkinje cells. Additionally, Purkinje cells from these knockout mice exhibit significantly reduced dendritic areas compared to wild-type cells in dissociated cultures [21]. Similarly, a point mutation in the Trpc3 protein, specifically T635A, has been shown to cause significant impairment in the growth and differentiation of Purkinje cell dendrites [22]. Additionally, abnormal dendritic development of Purkinje cells has been observed in cultured cerebellar cells from ITPR1 knockout mice [23,24]. Although no marked anatomical abnormalities were detected in the cerebellums of mGluR1-deficient mice, the Purkinje cell dendritic arbors were found to be smaller and less complex [25]. These findings suggest a potential link between SCA pathogenesis and disease-causing genes that mediate Purkinje cell development. SCAs are neurodegenerative disorders, and early mutations in genes encoding these proteins may contribute to the growth processes of Purkinje cells, ultimately leading to dysfunction in mature Purkinje cells, which remain present and alive in the cerebellum. Some researchers have posited that the influence of disease-causing genes on Purkinje cell development may determine the severity of the disease in adult SCA mouse models [26]. Consequently, our study seeks to investigate these potential relationships by employing dissociated Purkinje cell culture techniques to observe and analyze the dendritic growth of Purkinje cells.

In a previous investigation, we reported that mutations in PKCγ, specifically S361G, cause SCA14. In a mouse model of SCA14, the expression of mutated PKCγ-S361G led to dendritic abnormalities in Purkinje cells [18]. However, transgenic mice harboring the PKCγ-G118D mutation did not exhibit overt ataxia or abnormal dendritic growth in cerebellar Purkinje cell cultures. It is noteworthy that our earlier PKCγ-G118D mouse line featured concurrent expression of both the endogenous mouse PKCγ and the transgenic human PKCγ-G118D [18]. Quantitative PCR (qPCR) confirmed the expression of the human *PRKCG* transgene; however, total PKCγ protein levels in the C1 domain-mutated PKCγ-G118D line were notably lower, at approximately 52%, compared to a separate C3 domain-mutated transgenic line that manifested severe ataxia and Purkinje cell abnormalities [18]. Consequently, we could not conclusively rule out the possibility that insufficient transgene expression in the G118D mice hindered the emergence of a discernible phenotype. To address this limitation, we have now successfully integrated a Purkinje cell-specific expression vector with a novel PKCγ knockout mouse line, which lacks endogenous mouse PKCγ expression. The expression of the human *PRKCG* gene was verified using anti-PKCγ antibody. In dissociated cultures of cerebellar PKCγ knockout Purkinje cells, we observed a significant reduction in the mean dendritic area of Purkinje cells expressing PKCγ-G118D, down to 76.6% of that in control Purkinje cells. This finding underscores the potent inhibitory effect of the SCA14-associated PKCγ-G118D protein on dendritic growth. These results provide compelling evidence that PKCγ-G118D exerts a detrimental influence on the development of cerebellar Purkinje cells. Importantly, our study highlights that the PKCγ-G118D mutation alone is sufficient to impair dendritic morphogenesis in the absence of compensatory mechanisms, establishing its intrinsic pathogenicity in Purkinje cell dysfunction.

To delve deeper into the biological activity of PKCγ-G118D, we conducted transfection experiments in dissociated cerebellar cultures derived from a PKCγ knockout mouse line, using plasmids encoding both the SCA14-associated PKCγ-G118D mutant and human PKCγ-WT. This setup effectively eliminated any confounding influence from endogenous mouse PKCγ. Analogous to the effects observed in wild-type mouse Purkinje cells transfected with PKCγ-WT [18], our results revealed a modest, yet statistically insignificant, reduction in dendritic tree size upon PKCγ-WT overexpression in the PKCγ knockout cells. Conversely, transfection with PKCγ-G118D in these PKCγ-null Purkinje cells potently inhibited dendritic growth, underscoring its inhibitory potency. This stark contrast between WT and mutant PKCγ reinforces the notion that G118D disrupts critical regulatory checkpoints required for balanced kinase activity during dendritogenesis. Intriguingly, while PKCγ-G118D demonstrated elevated activity in vitro assays, akin to kinase domain mutations, its biological impact in Purkinje cells was mitigated in the presence of endogenous PKCγ in prior studies [18,19]. This attenuation could stem from mechanisms such as augmented autophosphorylation, compromised activation of downstream signaling pathways, or protein aggregation [14,17,18,19]. Notably, the pathological underpinnings of SCA14 may not be straightforwardly tied to the biological activity of the mutant per se, but rather a “loss of regulation” that plays a pivotal role in disease pathogenesis. Specifically, the inability of PKCγ-G118D to swiftly adapt its activity to the dynamic demands of Purkinje cells may lead to aberrant kinase activity, ultimately contributing to cell dysfunction and demise [18]. However, in the transgenic mouse line co-expressing both mouse PKCγ and human PKCγ-G118D protein, the presence of endogenous mouse PKCγ may serve as a compensatory mechanism, mitigating the “loss of regulation” of Purkinje cells. Such compensation could explain the phenotypic discrepancies between our knockout model and previous transgenic lines, emphasizing the importance of genetic background in modeling SCA14. Collectively, our findings underscore the contextual modulation of human PKCγ-G118D biological activity, revealing its capacity to negatively impact Purkinje cell growth in the absence of endogenous mouse PKCγ. This key conclusion positions PKCγ homeostasis as a critical factor in SCA14 progression and suggests that therapeutic strategies targeting PKCγ dysregulation, rather than mere activity levels, may hold promise.

The precise mechanisms underlying the muted effect of PKCγ-G118D in the presence of endogenous PKCγ remain enigmatic and warrant further investigation. Future studies could explore whether endogenous PKCγ stabilizes mutant protein turnover, modulates its subcellular localization, or rescues downstream signaling deficits—questions that could be addressed via live imaging or proteomic analyses. Furthermore, we propose that the development of a novel knock-in mouse line exclusively harboring the SCA14-assoicated PKCγ-G118D mutation could serve as a valuable tool for unraveling the intricacies of this mutant and other regulatory domain variants in the future. Such a model would circumvent overexpression artifacts and enable longitudinal assessment of how PKCγ-G118D alters cerebellar circuit formation and motor coordination, bridging the gap between cellular dysfunction and clinical manifestations.

## 4. Materials and Methods

### 4.1. Mice

The experiments conducted herein strictly adhered to the guidelines outlined in the EU Directive 2010/63/EU pertaining to the ethical treatment and utilization of laboratory animals. Prior to commencement, these experiments received the green light from the veterinary office of the canton of Basel and were further authorized by the relevant Swiss authorities. To establish primary mouse cerebellar cell cultures, CRISPR/Cas9 technology was employed to generate PKCγ knockout mice, which served as the experimental model. PKCγ knockout mice were obtained as a byproduct of the same experimental process of the generation of a PKCγ-A24E knock-in mice, which originated from the introduction of point mutations into the *PRKCG* gene located on chromosome 7, specifically c.71C > A (p.Ala24Glu) and c.78G > A (p.Arg26Arg) in FVB background mice. The c.78G > A mutation was intentionally introduced into the Protospacer Adjacent Motif (PAM) sequence to prevent the donor DNA from being a suitable target for Cas9 cleavage. The knock-in mice were created using the Cas9/CRISPR engineering system at the Center of Transgenic Models, University of Basel, employing the rapid oocyte injection method. Alt-R CRISPR-Cas9 crRNA, designed specifically to target exon 1 of *PRKCG* (sequence: T TGC AGA AAG GGG GCG CTG) upstream of the PAM sequence, along with Alt-R CRISPR-Cas9 tracrRNA and donor DNA, were obtained from Integrated DNA Technologies. The crRNA, Cas9, and donor DNA were injected into FVB zygotes at the pronuclear site, and surviving embryos were transferred into pseudo-pregnant mothers. To identify founders, genotyping was performed on genomic DNA samples obtained from biopsies using PCR. The primers used for genotyping were as follows: forward primer, 5′ TCC TTC CTA TCT CAG AGT CTG CG 3′; and reverse primer, 5′ GTT CCC AAG TCC CCT CCT TTT CC 3′ (Microsynth). The presence of the c.71C > A and c.78G > A mutations in the *PRKCG* gene was confirmed by DNA sequencing. The fragment for sequencing was obtained by PCR using genomic DNA samples and the aforementioned primers. However, during this process, we also identified mice with unintended *PRKCG* knockout, which were retained and analyzed separately as *PRKCG* knockout mice.

### 4.2. Plasmid Construction

The cloning process entailed integrating the SCA14-associated PKCγ-G118D mutant and the human PKCγ coding sequences into the pL7 vectors, specifically designed for targeted expression in Purkinje cells [18,20]. This was accomplished utilizing the EndoFree Plasmid Maxi Kit (QIAGEN, Hilden, Germany), adhering strictly to the manufacturer’s prescribed protocol. Prior to cloning, the human PKCγ sequence was amplified via PCR from the Origene pCMV6-XL4-PRKCG vector sourced from Rockville, MD, USA. Conversely, the SCA14-linked PKCγ-G118D mutant was generated through a site-directed mutagenesis PCR approach, leveraging the pCMV6-XL4-PRKCG vector as a template.

### 4.3. Cultures of Cerebellar Purkinje Cells and Electroporation Procedure

To prepare primary cultures of cerebellar Purkinje cells from PKCγ knockout mice, neonatal mice were utilized following a well-established protocol [20]. In summary, cerebella were extracted from mice at postnatal day 0, subsequently dissociated, and plated onto glass chambers pre-coated with Poly-D-lysine. The Neon Transfection System (Thermo Fisher Scientific, Waltham, MA, USA) was employed to target the introduction of the indicated vectors into Purkinje cells. To prepare for the transfection of dissociated Purkinje cells using the Neon Transfection System, first cultivate the required number of cells on the day of transfection. Then, prepare the cells by resuspending them in Resuspension Buffer R at a final density suitable for the electroporation volume for 10 µL tips. Simultaneously, prepare the plasmid DNA at a concentration of 1–5 µg/µL in TE buffer. Next, turn on the Neon device and navigate to the desired electroporation protocol input window with the specific parameters for Purkinje cells: a pulse voltage of 1200 V, a pulse width of 30 ms, and a single pulse. For the electroporation procedure, transfer the appropriate amount of plasmid DNA into a sterile microcentrifuge tube, add the resuspended cells to the tube containing the DNA, and gently mix. Load the cell-DNA mixture into a Neon tip by inserting the tip into the Neon pipette and aspirating the mixture. Insert the Neon pipette with the loaded tip into the Neon tube placed in the Neon pipette station, and initiate electroporation. After electroporation, immediately remove the Neon pipette and transfer the transfected cells into a pre-warmed culture plate containing the appropriate medium comprising 90% Dulbecco’s modified Eagle medium/F-12 nutrient-based medium, supplemented with 1% N2, 1% glutamax, and 10% FBS, all sourced from Life Technologies, Zug, Switzerland. While serum contains trace amounts of growth factors, we did not observe obvious compensatory dendritic growth in the study. Within 2–4 h post-transfection, each well received an addition of 500 μL of DFM enriched with 1% N2 and 1% glutamax. To ensure optimal culture conditions, half of the medium was replaced every 4 days. Ultimately, cells were maintained in culture for a period of 2 weeks prior to fixation.

### 4.4. Immunocytochemistry

The cerebellar Purkinje cells from the PKCγ knockout mouse were fixed in 4% paraformaldehyde for 30 min at room temperature. During the staining process, all reagents were diluted in a 100 mM phosphate buffer (PB) set to a pH of 7.3. After fixation, the cells were incubated with the primary antibody, diluted in a blocking solution composed of PB, 3% non-immune goat serum, and 0.5% Triton X-100 (Sigma, St. Louis, MO, USA), for 1 h at room temperature. The cells were then rinsed with PB and incubated with the respective fluorescence-labeled secondary antibodies, suspended in PB containing 0.1% Triton X-100, for 2 h at room temperature. The cells were mounted with Mowiol (Sigma-Aldrich, Buchs, Switzerland) following staining. The mounted cells were imaged using an Olympus AX-70 (Olympus Corporation, Tokyo, Japan) fluorescence microscope equipped with a Spot Insight digital camera. The primary antibodies used in this study included mouse anti-PKCγ (1:4000, Santa Cruz Biotechnology, Inc., Dallas, TX, USA), rabbit anti-Calbindin D-28K (1:500, Swant, Marly, Switzerland), Guinea pig anti-Calbindin (1:4000, SYSY), rabbit anti-GFP (1:2000, Novus, Zug, Switzerland), mouse anti-Calbindin D-28K (1:500, Swant, Marly, Switzerland), and chicken anti-GFP (1:2000, Abcam, Cambridge, UK). The secondary antibodies used in this study included anti-mouse Alexa 488, anti-guinea pig Alexa Fluor 568, anti-chicken Alexa Fluor Plus 488, and anti-rabbit Alexa 488, all diluted 1:2000 and sourced from Molecular Probes (Eugene, OR, USA).

### 4.5. Quantitative Analysis

The process of quantifying dendritic area was executed utilizing a previously described image analysis program, as referenced in the previous study [20]. This method initially established the average value of control Purkinje cells as the benchmark (set at 1). To ensure a parallel growth milieu, non-transfected Purkinje cells, positioned in close proximity to transfected cells identified by their unique tagging protein expression, were selected as the control group. Both cell types were subjected to identical transfection and culture conditions. Subsequently, the ImageJ software (version 1.53) was employed to meticulously trace the contours of the Purkinje cells, thereby enabling the determination of the total area encompassing the cell body and its dendritic arborization. The subsequent data analysis was conducted utilizing GraphPad Prism software (version 9.5.1), sourced from San Diego, CA, USA. Prior to analysis, the displayed images underwent linear adjustments in both brightness and contrast to optimize visualization. Statistical evaluation of the observed parameter variations was conducted through the application of the non-parametric Mann–Whitney test. The confidence intervals were set at 95%, with statistical significance being deemed present at a *p*-value threshold of <0.05.

## Data Availability

The raw data supporting the conclusions of this article will be made available by the authors on request.
